# Stereotactic body radiation therapy for early-stage non-small cell lung cancer: a single-institutional retrospective analysis of outcomes and prognostic factors

**DOI:** 10.3389/fonc.2025.1591420

**Published:** 2025-09-16

**Authors:** Chengrui Fu, Jigang Dong, Chunhui Li, Zhongtang Wang, Wei Huang, Chengxin Liu, Dan Han, Bin Zhang, Baosheng Li

**Affiliations:** ^1^ Tianjin Medical University Cancer Institute and Hospital, National Clinical Research Center for Cancer, Key Laboratory of Cancer Prevention and Therapy, Tianjin’s Clinical Research Center for Cancer, Tianjin, China; ^2^ Department of Radiation Oncology, Tianjin Medical University, Tianjin, China; ^3^ Department of Radiation Oncology, Shandong Cancer Hospital and Institute, Shandong First Medical University and Shandong Academy of Medical Sciences, Jinan, China; ^4^ Qingdao People’s Hospital Group (Jiaozhou), Jiaozhou Central Hospital of Qingdao, Qingdao, China

**Keywords:** stereotactic body radiotherapy, SBRT, early-stage NSCLC, lung cancer, prognostic factors

## Abstract

**Background:**

Stereotactic body radiotherapy (SBRT) is a definitive treatment for medically inoperable early-stage non-small cell lung cancer (NSCLC), yet optimal dose selection and prognostic factors in elderly, high-risk populations remain debated. This study evaluates long-term outcomes and predictors of survival in a real-world cohort.

**Methods:**

We retrospectively analyzed 258 patients with T1-2N0M0 NSCLC treated with SBRT at Shandong Cancer Hospital (2017–2022). Inclusion criteria: tumors ≤5 cm, medically inoperable or surgery-refused. Survival outcomes (LC, PFS, CSS, OS) were estimated using Kaplan-Meier curves with log-rank tests. Competing risk regression (Fine-Gray model) was used for cancer-specific survival (CSS), with non-cancer deaths as competing events. Prognostic factors of OS via univariable and multivariable Cox regression. Dose fractionation was individualized (median BED_10_=100 Gy, range: 75–144 Gy), with strict adherence to RTOG 0236 constraints, using 4D-CT for motion management and daily CBCT for image guidance.

**Results:**

The cohort comprised predominantly elderly patients (median age: 73 years; 41.5% ≥75 years, 21.3% ≥80 years). At a median follow-up of 38.8 months, 5-year OS, progression-free survival (PFS), local control (LC), and CSS rates were 74.2%, 71.9%, 83.8%, and 84.5% respectively. Competing risks analysis revealed cumulative 5-year cancer-specific mortality of 14.1% (7.6%–20.5%) versus non-cancer mortality of 11.6% (6.8%–16.4%). Multivariable analysis identified lower lobe lung cancer (HR = 2.218, *p* = 0.014), central tumor location (HR = 2.664, *p* = 0.003), the larger tumor length (HR = 1.415, *p* = 0.039), smoking history (HR = 2.328, *p* = 0.008) and medical inoperable (HR = 2.572, *p* = 0.007) as independent predictors of poor OS. Despite 21.3% central tumors, toxicity was minimal (grade 3 pneumonitis: 1.6%).

**Conclusion:**

SBRT achieves durable survival in early-stage NSCLC at our center. Central/lower lobe tumors, bigger tumors, smoking history, and medical inoperable independently predict inferior survival, emphasizing the need for personalized dose escalation strategies or combined treatment modalities.

## Introduction

Lung cancer remains the leading cause of cancer-related mortality worldwide, with non-small cell lung cancer (NSCLC) accounting for approximately 85% of cases (1). Advances in imaging technologies, particularly low-dose computed tomography (LDCT) screening, have increased the detection of early-stage NSCLC (clinical stage T1-2N0M0), for which curative-intent treatment is paramount. Historically, surgical resection, preferably video assisted surgery-VATS lobectomy, has been the gold standard, offering 5-year overall survival (OS) rates of 60–80% in operable patients. However, a significant proportion of patients-up to 30% in elderly populations-are deemed medically inoperable due to comorbidities such as chronic obstructive pulmonary disease (COPD) or cardiovascular disease (2), while others decline surgery due to personal preference or perceived risks.

Stereotactic body radiotherapy (SBRT), delivering ablative radiation doses with millimeter precision, has emerged as a standard alternative for inoperable early-stage NSCLC. Landmark trials, including JCOG0403 (3) and RTOG 0236 (4), established SBRT as a safe and effective modality, achieving local control rates exceeding 90%. Subsequent studies have further validated its role in high-risk surgical candidates, particularly those with central tumors or advanced age. However, some studies have shown a lower OS with SBRT than surgery for early-stage NSCLC (5). Prospective, randomized, controlled trials are needed to answer these questions because the findings are conflicting. Three-phase 3 randomized studies have been initiated to compare SBRT with surgery in patients with early-stage NSCLC (the STARS trial [NCT00840749], the ROSEL trial [NCT00687986], and the ACOSOG Z4099 trial [NCT01336894]), however, all were closed early because of slow accrual. Recent models, such as that proposed by Vanstraelen et al. (6), predict SBRT referral in operable patients and highlight long-term outcomes, underscoring the need for personalized selection in high-risk cohorts.

This retrospective study analyzes a single-institutional cohort of early-stage NSCLC patients treated with SBRT. We aim to: 1. evaluate 5-year survival outcomes in a real-world elderly cohort, including cancer-specific survival (CSS) and competing mortality risks; 2. analyze dose-response relationships across heterogeneous fractionation schemes; and 3. identify clinical and dosimetric predictors of survival. By integrating competing risk models and multivariable analyses, this work addresses the interplay between tumor biology and treatment parameters-a critical step toward personalized SBRT strategies in an era of increasing geriatric and medically complex NSCLC populations.

## Methods

### Study design and population

**Figure 1:**
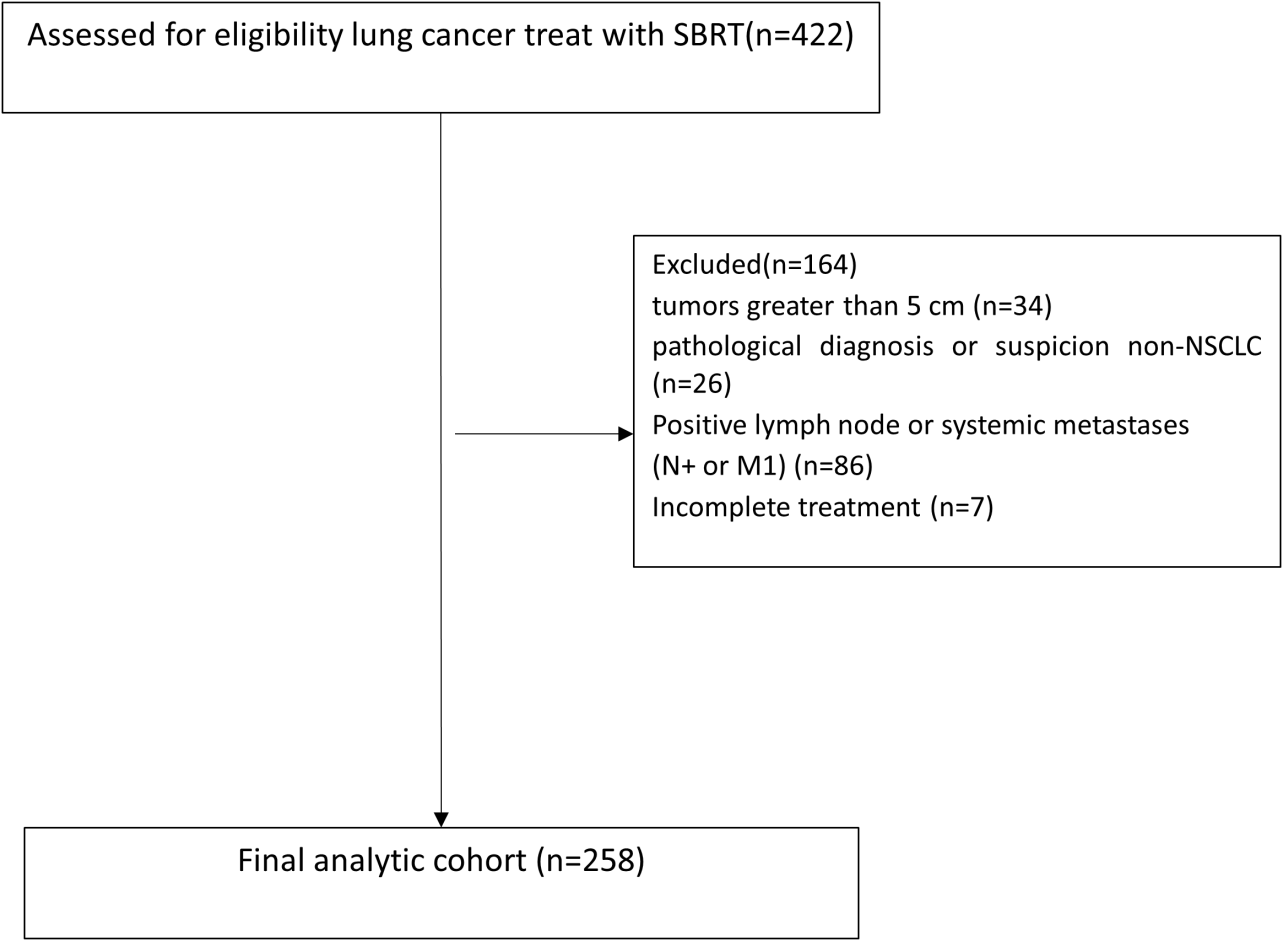
CONSORT diagram for exclusions.

Clinical data were collected from patients with early-stage (T1-2N0M0) lung cancer who underwent SBRT at Shandong Cancer Hospital between June 2017 and July 2022. SBRT was defined as≥5 Gy per fraction using photons, in case of hypercentral lung cancer, 60Gy/15fractions, 4Gy per fraction was also permissible. Tumors were staged according to the 8th edition of the American Joint Committee on Cancer (AJCC) TNM staging system (7). All patients were discussed as either ineligible for surgery or refused surgery and were scheduled for SBRT by radiation oncologist. Inclusion criteria: 1. tumor diameter ≤5 cm (T1: ≤3 cm, T2: >3–5 cm); 2. no evidence of nodal or distant metastasis (staging via PET-CT and/or brain MRI if necessity); 3. medically inoperable or refusal of surgery. Exclusion criteria were as follows: 1. tumors greater than 5 cm in the greatest dimension; 2. patients with pathological diagnosis or suspicion of small cell lung cancer (SCLC) or large cell neuroendocrine carcinoma (LCNEC); 3. positive or suspected regional lymph node metastases, mediastinal spread, or systemic metastases inferred by CT, PET-CT, or endobronchial ultrasound (EBUS); 4. 1 month or less of follow-up; 5. patients with missing treatment information; and 6. treatment was not completed as planned. Patient flow diagram was shown in **Figure 1**.

All cases were pathologically or radiologically confirmed. Biopsy was avoided if contraindicated (e.g., central location, poor PS). For the 31% of patients without histological confirmation, SBRT was initiated after multidisciplinary team consensus. Clinical-radiological criteria included FDG-PET positivity (performed in 52% of cases) and/or progressive lesion enlargement on serial CT imaging (observed in 37.5%). The following parameters were systematically documented. Demographics: gender, age, smoking history, and comorbidities. Poor pulmonary function: FEV1 <50% predicted/DLCO<40% predicted, per ATS guidelines or other clinical indicators when inability to perform spirometry, such as Breath-Holding Test <20s, Stair-Climbing Test <2 floors and 6-minute walking distance (6MWD): <300 meters. Tumor characteristics: location (left/right, upper/middle/lower lobe, peripheral/central), size (maximum diameter in cm), histopathological subtype (if biopsy-confirmed). Definitive diagnostic basis (pathology vs. radiology). Treatment details: reasons for non-surgical management (medical inoperability vs. patient refusal), radiation dose per fraction (Gy), total number of fractions, and treatment schedule (daily vs. every-other-day delivery). Concurrent or adjuvant therapies (e.g., immunotherapy, chemotherapy, targeted therapy). The treatment-related toxicity was assessed according to the Common Terminology Criteria for Adverse Events (CTCAE), version 5.0.

### Follow-up

In principle, follow-up chest CT scans were performed after the completion of SBRT every 3 to 6 months for the first two years, every 6 months for the next 2–5 years, and annually thereafter, unless the patient refused or other obstacles. Other follow-up items included interviews, laboratory data review, B-ultrasonography or CT scans of the abdomen. Brain magnetic resonance imaging (MRI), bone emission computed tomography (ECT), and FDG-PET were also used if needed. The follow-up duration was measured from the first day of SBRT to death or the date of the last follow-up. Events of interest included local control (LC), progression-free survival (PFS), CSS, and OS, which were calculated from the starting day of SBRT until the occurrence of an event of interest, or death or last follow-up. Pattern of progression (local recurrence, regional nodal involvement, distant metastasis), and cause of death (cancer-related or other reasons) should register clearly during the follow-up visit.

### SBRT treatment details

The gross tumor volume (GTV) was delineated as a lesion observed at the lung window level on the enhanced CT and/or FDG-PET. The clinical target volume was equal to GTV. The internal target volume (ITV) was contoured based on the extension of GTVs at the all phases (5 inspiratory, 5 expiratory, and 1 resting phase) of the respiratory cycle on the four dimensional CT (4D-CT) (Siemens Somatom Sensation, Siemens Healthineers Corporation, Germany) scanning to include the full movement of the tumor.

To compensate for the uncertainty in tumor position and changes in tumor motion caused by breathing, the planning target volume (PTV) was extended by a margin of 0.5-0.6 cm from the ITV. Cone beam CT was implemented before each treatment to confirm the position of the target was achieved. The main factors determining the dose/fractionation scheme were tumor location, tumor size, and lung function parameters. In the setting of heterogeneous dose fractionation schedules, doses were converted to biologically effective dose (BED) using the following formula: BED_α/β_ = nd (1 + d/(α/β)) where n is the total number of fractions, d is the dose per fraction, and α/β is the alpha/beta ratio of the tumor (α/β = 10). Dose constraints for the OARs were implemented according to the experience of the Radiation Therapy Oncology Group (RTOG) 0236 guidelines (8).

### Statistical analyses

Continuous variables are presented as median and range. Categorical variables are presented as frequencies and percentages. Differences in treatment outcomes, including LC, PFS and OS, were calculated using Kaplan-Meier survival curves with log-rank tests. Univariable/multivariable Cox regression analysis for OS. Multivariable Cox proportional hazards regression used forward likelihood ratio (LR) selection (entry p <0.05) for variables significant on univariable analysis. Variables with p<0.05 in univariate analysis were entered. Some significant variables (e.g., sex, T stage, histology, and radiation fractions) were excluded stepwise due to multicollinearity, as male sex strongly overlapped with smoking status, tumor length correlated with T stage, and adenocarcinoma predominated in peripheral tumors. Competing risks (Fine-Gray) were used for CSS (cancer vs. non-cancer death). Deaths from causes unrelated to lung cancer were treated as competing events. Statistical analyses were performed with SPSS statistical software, version 29.0 and R statistical software, version 4.4.2. A p value less than 0.05 was considered statistically significant.

## Results

### Patient and treatment characteristics

From 2017 to 2022, a total of 258 eligible patients were collected. The median age of these patients was 73 years (range: 49–85), with 41.5% aged ≥75 years and 21.3% aged ≥80 years, reflecting an overall elderly population. 58.5% patients were medically inoperable and 41.5% patients refusing surgery. Tumor size and location: T1 (69.8%), T2 (30.2%); peripheral (78.7%), central (21.3%). Among 258 patients, 178 (69.0%) had pathological confirmation (132 adenocarcinoma, 46 squamous cell carcinoma), while 80 (31.0%) were diagnosed based on multidisciplinary consensus integrating imaging and clinical criteria. 151(58.5%)patients could not tolerate surgery, and 107 (41.5%) patients refused surgery due to advanced age or surgical risks. Demographic and clinical characteristics are shown in **Table 1**.

**Table 1:** Patients and treatment characteristics (N=258).

Characteristics	Overall N (%)	Inoperable N (%)	Refused surgery N (%)
Age (years)			
Median (range)	73 (49-85)	74 (53-85)	72 (49-82)
Sex			
Male	158 (61.2)	99 (62.7)	59 (37.3)
Female	100 (38.8)	52 (52.0)	48 (48.0)
Comorbidities			
cardiac conditions	93 (36.0)	67 (72.0)	26 (28.0)
poor pulmonary function	63 (24.4)	55 (87.3)	8 (12.7)
pulmonary surgery history	28 (10.9)	27 (96.4)	1 (3.6)
cerebrovascular diseases	34 (13.2)	17 (50.0)	17 (50.0)
others malignancies	51 (19.8)	47 (92.2)	4 (7.8)
Smoking index			
Smoking history	111 (43.0)	67 (60.4)	44 (39.6)
Median (range)	0 (0-2720)	0 (0-2720)	0 (0-2000)
Tumor location			
Left lung	116 (45.0)	69 (59.5)	47 (40.5)
Right lung	142 (55.0)	82 (57.7)	60 (42.3)
Upper lobe	148 (57.4)	85 (57.4)	63 (42.6)
Middle lobe	15 (5.8)	8 (53.3)	7 (46.7)
Lower lobe	95 (36.8)	58 (61.1)	37 (38.9)
Peripheral	203 (78.7)	124 (61.1)	79 (38.9)
Central	55 (21.3)	27 (49.1)	28 (50.9)
Tumor length(cm)			
Median (range)	2.15 (0.7-4.7)	2.0 (0.7-4.5)	2.2 (0.7-4.7)
Tumor nature			
Ground glass nodule	27 (10.5)	16 (59.3)	11 (40.7)
Solid tumor	179 (69.4)	113 (63.1)	66 (36.9)
Mixed ground glass nodule	52 (20.2)	22 (42.3)	30 (57.7)
T stage			
T1	180 (69.8)	107 (59.4)	73 (40.6)
T2	78 (30.2)	44 (56.4)	34 (43.6)
Pathology			
Adenocarcinoma	132 (51.2)	73 (55.3)	59 (44.7)
Squamous cell carcinoma	46 (17.8)	22 (47.8)	24 (52.2)
Not available	80 (31.0)	56 (70.0)	24 (30.0)
Reasons for not Operating			
Inoperable	151 (58.5)	/	/
Reject	107 (41.5)	/	/
Radiation dose per fraction (Gy)			
Median (range)	7 (4-11)	7 (4-11)	7.5 (4-11)
Radiation fractions			
Median (range)	8 (5-17)	8 (5-15)	8 (5-17)
Treatment schedule			
Daily	223 (86.4)	128 (57.1)	96 (42.9)
Every-other-day	34 (13.2)	23 (67.6)	11 (32.4)
Other treatments			
Chemotherapy	11 (4.3)	7 (63.6)	4 (36.4)
Targeted therapy	18 (7.0)	9 (50.0)	9 (50.0)
Immunotherapy	25 (9.7)	7 (28.0)	18 (72.0)
None	204 (79.1)	128 (62.7)	76 (37.3)

For continuous variables, the median and range are given; for categorical variables, the number of patients and percentages are given.

### SBRT treatment protocol

The single dose of radiotherapy was 4-11Gy, the median value was 7Gy, the fractions of radiotherapy range from 5 to 15, the BED_10_ range from 75Gy to 144Gy, the median BED_10_ value was 100Gy, and 56.2% of the BED_10_ was greater than or equal to 100Gy. The Common Dose Fractionation Schemes are shown in **Table 2**. Radiation oncologists individualize radiation doses and fractionation schedules based on factors such as lesion size, number, proximity to the chest wall and main bronchi, respiratory motion characteristics, and tumor location. Additionally, they determine whether to deliver treatment daily or every-other-day to optimize therapeutic efficacy while minimizing toxicity. All treatments utilized cone-beam CT (CBCT) for daily image guidance, with respiratory motion managed by 4D-CT simulation in 98% of cases.

**Table 2:** Common dose fractionation schemes (n=219, 89.15% of the cohort).

Dose (Gy)	Fractions	BED_10_ (Gy)	Number of cases	Proportion (%)
5	10	75	27	10.47
5	12	90	26	10.08
6	10	96	22	8.53
7	10	119	16	6.20
7~8	8	95.2-115.2	33	12.79
8	7	100.8	29	15.50
10~11	5	100-115.5	53	20.54
10	6	120	13	5.04

Represents predominant schemes; remaining 10.85% used less common regimens.

### Toxicity

Overall, the toxic effects were mild and well tolerated. Grade 1–2 pneumonitis occurred in 3.9% (n=10), while only 4 patients (1.6%) developed grade 3 pneumonitis, other grade 3 toxicities contained rib fracture (0.8%), esophagitis(1.16%). No grade 4–5 toxicities were observed.

### Survival outcomes

**Figure 2:**
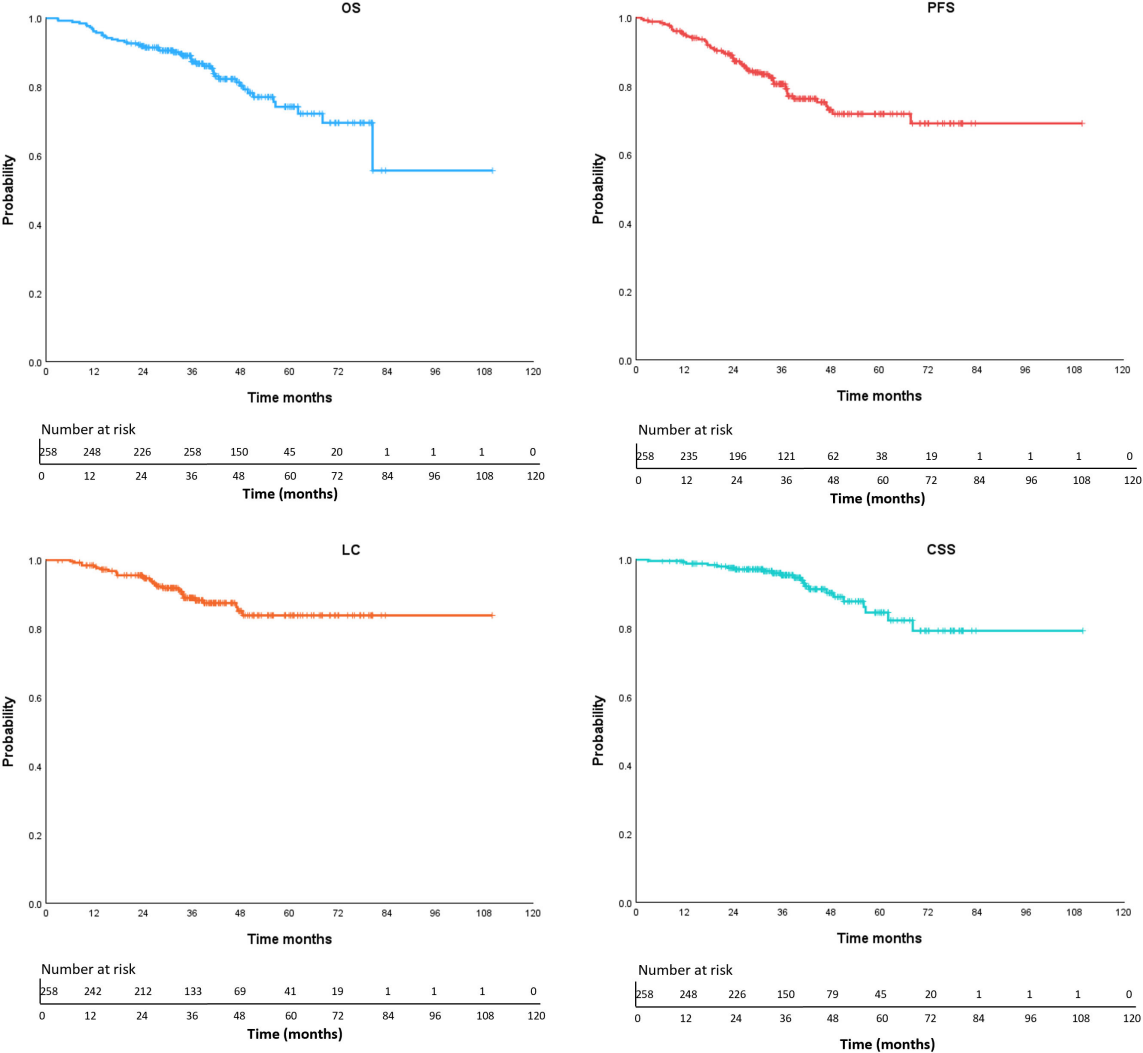
Kaplan-Meier curves of OS (overall survival), PFS (progression free survival), LC (local control), CSS (cancer-specific survival).

The final follow-up was conducted on October 21, 2024, with a median follow-up duration of 38.8 months (range: 2.9-109.9 months). A total of 55 patients (21.3%) experienced disease progression, including 14 cases (5.4%) of local recurrence only, 26 cases (10.1%) of distant metastasis only, and 15 cases (5.8%) with both local recurrence and distant metastasis. The 5-year local control rate was 83.8%. Among the 47 deceased patients (18.2%), 8.5% (22 patients) died due to tumor-related causes, while 8.1% (21 patients) died from non-tumor-related causes, including COVID-19 (5 patients) and other underlying conditions such as cardiovascular diseases. The cause of death remained undetermined in 4 cases (1.6%). The median PFS and overall survival (OS) were not reached. The 1-, 3-, and 5-year OS rates were 96.1%, 87.3%, and 74.2%, respectively. The 1-, 3-, and 5-year PFS rates were 94.9%, 80.6%, and 71.9%, respectively. The corresponding 1-, 3-, and 5-year LC rates were 98.0%, 88.9%, and 83.8%, respectively. The 1-, 3-, and 5-year CSS rates were 99.2%, 95.4%, and 84.5%, respectively (**Figure 2**). Competing risks analysis revealed a 5-year cumulative incidence of cancer-specific death of 14.1% (95% CI: 7.6% - 20.5%) versus 11.6% (95% CI: 6.8% - 16.4%) for non-cancer mortality.

**Figure 3:**
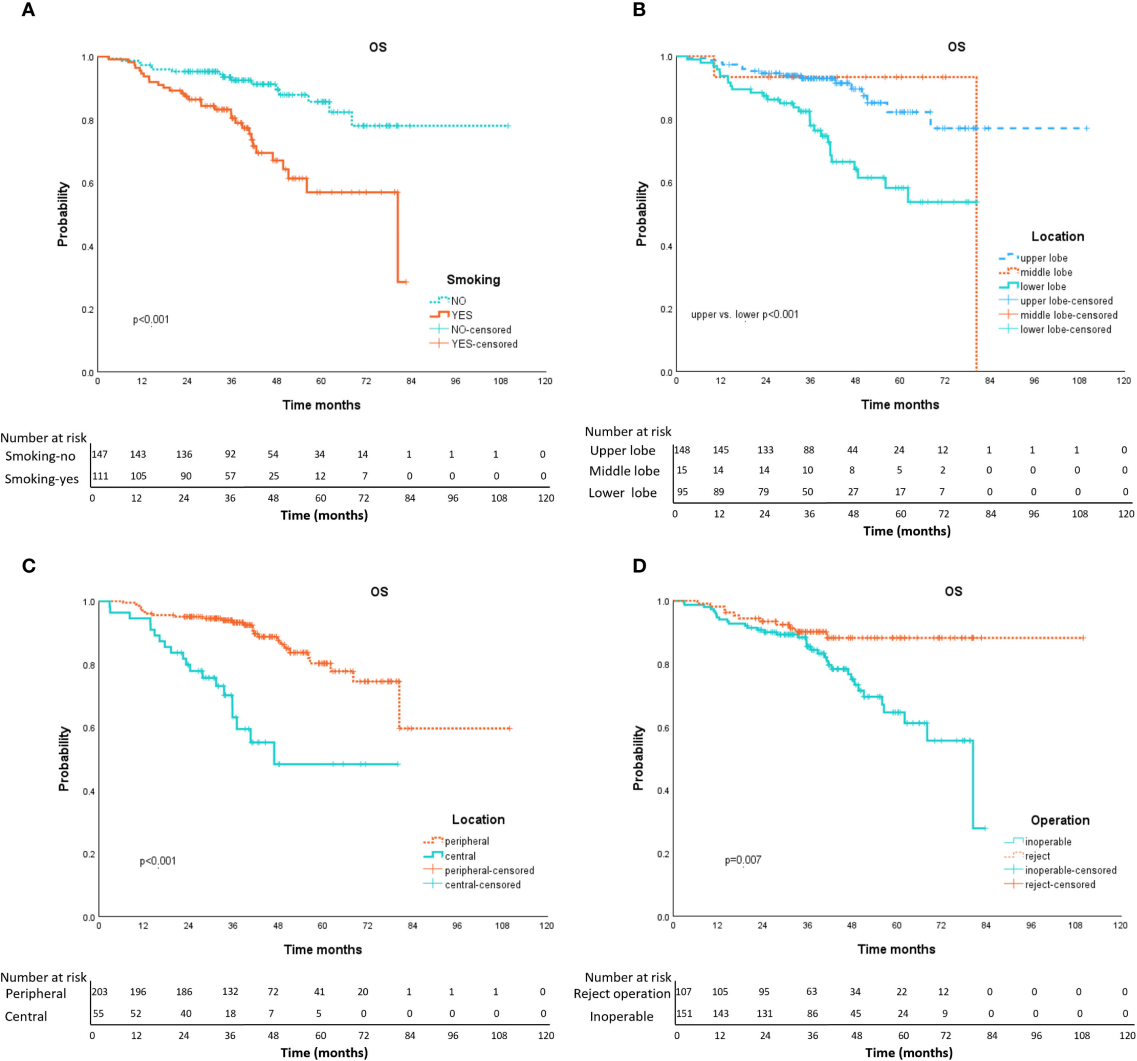
Kaplan-Meier curves of OS with variables from univariable analyses. Panel **(A)** smoking history (yes vs. no); Panel **(B)** tumor location (upper vs. lower); Panel **(C)** tumor location (peripheral vs. central); Panel **(D)** operation (inoperable vs. reject operate).

Univariate analysis (**Table 3**) showed that, age, left/right lobe, radiation schedule daily/every-other-day, and combination therapy were not significant correlation with OS. Gender, smoking history (**Figure 3A**), location of the lesion [upper/lower lobe (**Figure 3B**), peripheral/central (**Figure 3C**)], the nature of tumor, inoperable/reject operation (**Figure 3D**), pathology, tumor length, T stage, and radiation dose per fraction, radiation fractions, and BED_10_ were factors impact OS by univariable analysis. While multivariable COX regression analysis (**Table 3**) showed that lower lobe lung cancer (HR = 2.218, 95% CI 1.176–4.181, *p* = 0.014), central tumor location (HR = 2.664, 95% CI 1.394–5.091, *p* = 0.003), the larger tumor length (HR = 1.415, 95% CI 1.017-1.967, *p* = 0.039), smoking history (HR = 2.328, 1.243–4.358, *p* = 0.008) and medical inoperable (HR = 2.572, 1.300-5.091, *p* = 0.007) were significant negative prognostic factors of OS.

**Table 3:** Univariate and multivariate analyses affecting overall survival.

Variables	Univariate analysis			Multivariate analysis		
	HR	95% CI	P value	HR	95% CI	P value
Sex						
Female	1					
Male	2.666	1.35-5.266	**0.005**			
Age	1.017	0.977-1.059	0.400			
<75	1					
≥75	0.923	0.513-1.660	0.790			
Smoking	1.001	1.000-1.001	<0.001			
No	1					
Yes	3.164	1.725-5.804	**<0.001**	2.328	1.243-4.358	**0.008**
Tumor location						
Left lung	1					
Right lung	0.755	0.425-1.340	0.337			
Upper lobe	1					
Middle lobe	0.965	0.221-4.215	0.962	1.072	0.238-4.836	0.928
Lower lobe	3.093	1.679-5.699	**<0.001**	2.218	1.176-4.181	**0.014**
Peripheral	1					
Central	4.312	2.380-7.813	**<0.001**	2.664	1.394-5.091	**0.003**
Tumor length	1.730	1.272-2.353	<0.001	1.415	1.017-1.967	0.039
Tumor nature						
GGN	1					
Solid tumor	8.332	1.146-60.580	**0.036**			
Mixed GGN	1.519	0.137-16.817	0.733			
T stage						
T1	1					
T2	2.239	1.3111-4.135	**0.004**			
Pathology						
AC	1					
SCC	3.287	1.688-6.403	**<0.001**			
Not available	0.809	0.388-1.690	0.574			
Operation						
Reject	1					
Inoperable	2.526	1.283-4.971	**0.007**	2.572	1.300-5.091	**0.007**
BED_10_	0.966	0.946-0.986	0.001			
≥100Gy	1					
<100Gy	2.315	1.289-4.156	**0.005**			
RT dose/F(Gy)	0.753	0.638-0.889	<0.001			
RT fractions	1.180	1.061-1.312	0.002			
RT schedule						
Daily	1					
Every-other-day	0.800	0.316-2.025	0.638			
Other treatment						
None	1					
Chemotherapy	1.990	0.709-5.590	0.191			
Targeted therapy	0.821	0.253-2.667	0.743			
Immunotherapy	0.585	0.140-2.442	0.462			

GGN, Ground glass nodule; AC, adenocarcinoma; SCC, squamous cell carcinoma. Variables significant in univariate analysis but not retained in the multivariable model were excluded due to multicollinearity (e.g., sex vs. smoking, tumor length vs. T stage, tumor location vs. histology). Bold values indicate statistical significance (p<0.05)

Among the 14 patients with isolated local recurrence, 3 (21.4%) underwent salvage SBRT at a median interval of 27.3 months (range: 11.8–33.8 months) after initial treatment. At the last follow-up, none experienced secondary progression (PFS time: 13.8-28.3 months). Alternative interventions included surgery (n=1), radiofrequency ablation (n=1), brachytherapy (n=1), conventional radiotherapy (n=2), systemic therapy (n=3), or undocumented management (n=2).

## Discussion

SBRT is now the standard-of-care for medically inoperable early-stage NSCLC, due to its high local control rate and mild side effects especially in elderly patients, yet its role in operable patients remains debated. At our institution, approximately 2,500 lung cancer surgeries are performed annually. Based on epidemiological and multicenter data indicating that ~80% are NSCLC and ~66% of these are stage I–IIA (T1-2N0M0) (9), we estimate ~1,300 early-stage surgical cases per year. During the study period, 258 patients were treated with SBRT, representing approximately 4% of the potential early-stage cohort. This contextualizes the proportion of patients referred to SBRT in our practice.

In this retrospective analysis of 258 patients with early-stage NSCLC treated with SBRT at our institution, we observed robust long-term outcomes, including 5-year OS of 74.2%, PFS of 71.9%, LC of 83.8%, and CSS of 84.5%. Competing risks analysis highlighted a 5-year cancer-specific mortality of 14.1% (95% CI: 7.6%–20.5%) versus non-cancer mortality of 11.6% (95% CI: 6.8%–16.4%), underscoring the significant role of comorbidities in this elderly cohort (median age 73 years, 41.5% ≥75 years, 21.3% ≥80 years). Multivariable Cox regression identified independent predictors of inferior OS, including lower lobe location (HR = 2.218, *p* = 0.014), central tumor (HR = 2.664, *p* = 0.003), larger tumor diameter (HR = 1.415, *p* = 0.039), smoking history (HR = 2.328, *p* = 0.008), and medical inoperability (HR = 2.572, *p* = 0.007). Despite including 21.3% central tumors, treatment-related toxicity remained minimal, with grade 3 pneumonitis in only 1.6% of cases. Our 5-year OS rate of 74.2% compares favorably to historical SBRT studies (10–12),landmark trials such as RTOG 0236(4,12) (5-year OS 40% in medically inoperable patients) and JCOG0403 (3)(5-year OS 54% in operable patients), potentially attributable to our cohort’s mix of inoperable (58.5%) and surgery-refusal (41.5%) patients and lower overall comorbidity burden in the refusal subgroup. Routine 4D-CT for respiratory motion management (13) and daily CBCT for precise image guidance, enhancing tumor targeting, local control, and minimizing toxicity, as evidenced by improved outcomes in SBRT literature (14, 15). Subgroup analysis revealed superior OS in the refusal group (HR = 0.389, 95% CI: 0.196-0.773, p=0.007, adjusted for age and tumor factors), supporting the notion that fitter patients opting out of surgery contribute to better outcomes.

A recent predictive model by Vanstraelen et al. (6) for treatment selection in stage I NSCLC identified key referral predictors for SBRT over minimally invasive surgery (MIS), including advanced age, poor performance status, prior pulmonary resection, higher MSK-Frailty score, and reduced lung function, achieving an AUC of 0.908. This emphasizes clinical factors like age and comorbidities as primary drivers of SBRT referral in operable candidates. In contrast, our cohort’s high surgery-refusal rate (41.5%) highlights the influence of patient preference beyond these clinical predictors, particularly in an elderly Chinese population where cultural perceptions of surgical risks-such as fear of invasiveness, recovery challenges, and family decision making-may outweigh objective frailty metrics. This refusal rate is higher than in Western cohorts (typically 10-20%, up to 30% refusal rates in elderly populations (16–18)), reflecting regional differences in treatment attitudes and access to non-invasive options in China. Our study identified lower lobe lung cancer, central tumor location, the larger tumor length, smoking history and medical inoperable as independent predictors of inferior overall survival in early-stage NSCLC patients treated with SBRT, even after adjusting for confounding variables. The association between smoking and early death is well established (19). Compared with those who have never smoked, smokers have at least 15 times the risk of death from lung cancer and lose at least 1 decade of life expectancy (20). Research findings by Michael et al. have shown that OS is significantly improved in patients who stop smoking after SBRT for early-stage NSCLC (21).

The interplay between tumor location, dose, and outcomes remains pivotal. While our 5-year LC rate of 83.8% appears lower than the 98% reported in RTOG 0236 (12), this discrepancy likely stems from our inclusion of higher-risk subgroups: 21.3% central tumors and 30.2% T2 lesions-populations underrepresented in prior trials.

Central tumors’ proximity to critical structures (e.g., proximal airways, mediastinum) necessitated conservative fractionation (median BED_10_=81.25 Gy vs. 100.8 Gy for peripheral tumors, *p*<0.001). Such dose de-escalation, while reducing toxicity, may have contributed to the observed lower 3-year local control (75.1% vs. 91.6% in peripheral tumors). Contrasting with the EORTC LungTech trial (22), where 8 × 7.5 Gy yielded comparable LC but higher grade ≥3 toxicity (19.4%). For centrally located tumors, we should increase the prescribed dose or explore the combination therapy mode on the basis of controlling the side effects to further improve the local control rate and overall survival.

Compared with inoperable groups, patients who refused surgery had a better OS (3-year OS rate 90.1% vs 85.4%, 5-year OS rate 88.2% vs 64.6%), outcomes approaching surgical benchmarks in high-risk cohorts [e.g., JCOG 0802: 91.1% OS for lobectomy (23)], suggesting that SBRT treatment can also be chosen as an alternative for operable patients. Ackerson et al. found that SBRT and sublobar resection provided similar rates of local tumor control and overall clinical outcomes in stage I NSCLC (24).

Notably, although BED_10_ ≥100 Gy correlated with improved OS in univariable analysis (*p* = 0.005), its prognostic significance was attenuated in multivariable models-a phenomenon also observed in the HILUS trial (25), where ultra-central tumors achieved 80% LC with BED_10_=85.5 Gy. These findings suggest that tumor location may supersede dose escalation in determining outcomes, underscoring the need for location-specific protocols.

Non-cancer mortality accounted for a very important mortality risk in our cohort (11.6% vs. 14.1% cancer-specific mortality), consistent with the STARS trial (26) where 40% of deaths were unrelated to NSCLC. This pattern reflects the advanced age (median 73 years) and high comorbidity burden (36% cardiac disease, 24% pulmonary dysfunction) of SBRT cohorts, emphasizing the imperative for integrated comorbidity management (e.g., cardiopulmonary rehabilitation) alongside radiotherapy.

The favorable toxicity profile of SBRT further solidifies its role in frail populations. Grade ≥3 pneumonitis occurred in only 1.6% of patients-lower than rates reported for central tumors in RTOG 0813 [9.2% (27)]-with no grade 4–5 events. This safety advantage extends beyond surgery: a meta-analysis of high-risk surgical candidates reported 30-day mortality of 7–25% versus 0% with SBRT (28). It can be observed that the life quality has declined after surgery, studies after SBRT in mostly unfit patients showed no such decreases (29, 30). Radiofrequency ablation has also been evaluated for the treatment of NSCLC, but evidence suggests that local recurrence rates (25-35% vs. 10-15%), acute toxicity, and mortality in high-risk patients are higher with radiofrequency ablation than with SBRT (31), supporting SBRT as the preferred non-surgical modality.

All 3 patients in our study who received salvage SBRT achieved durable disease control, indicating that re-SBRT has application potential in patients with local recurrence, which is consistent with the findings of previous studies (32, 33). For selected cases, re-SBRT can be considered as a safe salvage strategy, careful patient selection (e.g., recurrence interval >12 months, small tumor volume) appears crucial to mitigate cumulative toxicity risks. Prospective studies are needed in the future to verify its long-term safety and dose accumulation effects.

This study has several limitations. First, its retrospective and single-institutional nature may introduce selection bias and limit the generalizability of our findings. Second, the median follow-up time was relatively short (38.8 months), largely due to the high proportion of patients treated in the later years of the study period (2021–2022). This restricts the maturity of long-term outcome data. Third, approximately one-third of patients were treated without histological confirmation. Although these cases were carefully reviewed in multidisciplinary tumor boards and decisions were based on PET-CT findings and/or progressive growth on serial imaging, the absence of tissue diagnosis remains a limitation compared with pathologically confirmed cohorts. Fourth, standardized pulmonary function data (FEV1, DLCO) were unavailable for many patients because most were referrals from outside institutions or were unable to perform spirometry due to advanced age or poor compliance. This may have introduced bias, as patients with the most impaired lung function may be underrepresented in our dataset. Nonetheless, alternative functional assessments such as stair-climbing, breath-holding, and six-minute walking tests were used to ensure clinical suitability for SBRT. Taken together, these limitations should be considered when interpreting the results. Despite these caveats, the study reflects real-world practice in a high-volume center and provides clinically relevant data on SBRT for early-stage NSCLC in the elderly population. Prospective trials such as VALOR (NCT02984761) are poised to clarify SBRT’s role in operable patients, while biomarker-driven studies may refine dose personalization. Phase II clinical trial suggest that SBRT combined with immunotherapy shows a beneficial trend compared with SBRT (34). There is currently a lack of high-level evidence to further improve the efficacy, and SBRT combined with immunotherapy is also a future research direction.

## Conclusion

In this real-world cohort, SBRT achieves durable survival in high-risk early-stage NSCLC, with outcomes better than other SBRT studies in elderly populations. Our data reinforce ASTRO guidelines recommending SBRT as first-line therapy for inoperable early-stage NSCLC (8). Lower lobe/central tumors, larger tumor length, smoking history, and medical inoperable independently predict inferior survival, these findings suggest that while SBRT is a highly effective treatment for early-stage NSCLC, further research is needed to refine treatment protocols for specific subgroups. Advanced imaging, biomarkers, combination treatment strategies and treatment planning techniques could help personalize SBRT and improve outcomes in challenging cases. Given the higher competing mortality risks in comorbid populations, the management of coexisting conditions also requires attention.

## Data Availability

The raw data supporting the conclusions of this article will be made available by the authors, without undue reservation.
